# *Latilactobacillus sakei* Wikim0066 Protects Skin through MMP Regulation on UVB-Irradiated In Vitro and In Vivo Model

**DOI:** 10.3390/nu15030726

**Published:** 2023-02-01

**Authors:** Jeong-Yong Park, Ji Yeon Lee, YongGyeong Kim, Chang-Ho Kang

**Affiliations:** MEDIOGEN, Co., Ltd., Biovalley 1-ro, Jecheon-si 27159, Republic of Korea

**Keywords:** wrinkle, ultraviolet B, collagen, matrix metalloproteinase, *Latilactobacillus sakei*

## Abstract

Ultraviolet (UV) B exposure induces wrinkle formation, collagen fiber breakdown, and transepidermal water loss (TEWL). UVB irradiation induces the expression of mitogen-activated protein kinase (MAPK), activator protein 1 (AP-1), and nuclear factor kappa B (NF-κB), which affect the expression of matrix metalloproteinases (MMP). We confirmed the effects of *Latilactobacillus sakei* wikim0066 (wikim0066) on UVB-irradiated Hs68 cells and HR-1 hairless mice cells. wikim0066 restored the production of type I procollagen by regulating the expression of MMP-1 and -3, MAPK, AP-1, and NF-κB in UVB-irradiated Hs68 cells and HR-1 mice. Oral administration of wikim0066 alleviates wrinkle formation, epidermal thickness, and TEWL in UVB-irradiated HR-1 hairless mice. These results indicated that wikim0066 has the potential to prevent UVB-induced wrinkle formation.

## 1. Introduction

Skin aging is characterized by the loss of structural and physiological skin functions and is classified as extrinsic and intrinsic aging [1]. Intrinsic aging is manifested by a decrease in the biosynthesis of biocomponent proteins and hormone secretion [2]. On the other hand, extrinsic aging is caused by photoaging caused by polluted air or ultraviolet (UV) rays [3]. Exposure to UVB causes DNA damage and destruction of the protein structure in the skin cells, resulting in wrinkle formation, inflammation, and cancer in the skin [4].

Wrinkle formation is caused by the degradation of ECM and the increase in matrix metalloproteinases (MMPs) [5]. Degradation of type I collagen, the most abundant protein in the ECM and skin connective tissue, increases during skin aging [6]. MMP, which contributes to wrinkling formation through ECM and collagen degradation, is activated by the mitogen-activated protein kinase (MAPK), activator protein 1 (AP-1), and nuclear factor kappa B (NF-κB) p65 pathways, which are enhanced by UVB irradiation [7,8]. In addition, the NF-κB pathway and MMP expression upregulate inflammatory cytokines such as interleukin (IL)-1β and IL-6 in UVB-irradiated skin [8,9]. Recent studies have reported that some compounds, such as polyphenols, retinyl palmitate, and hydroquinone, can improve skin aging, but they are expensive and have side effects [10,11]. Therefore, finding safe alternative agents is important to improve skin photoaging.

Probiotics are living microorganisms that, when ingested, improve host gut health [12]. Considerable evidence has been reported that probiotics can affect other organs by improving the composition of the gut microbiome [13,14]. Recent studies reported that probiotics could modulate the immune system and reduce inflammatory cytokines levels in the skin [15,16,17]. In particular, the *Lactobacillaceae* family exhibits various biological effects [18]. *Latilactobacillus sakei* is classified under the genus *Lactobacillaceae*, which is considered an important contributor to the elimination of pathogens through its antibacterial metabolites, intestinal barrier function, and immune responses [19]. *Latilactobacillus sakei* exhibits anti-inflammatory effects on the skin, such as atopic dermatitis and psoriasis, both in vitro and in vivo [20]. In addition, *Latilactobacillus sakei* suppresses MMP-1 by reducing the tumor necrosis factor-alpha (TNF-α), MAPK, and NF-κB signaling pathways in UVA-irradiated human dermal fibroblasts [21]. It has been reported that these effects result from cell-free supernatant (CFS) of the probiotics including short-chain fatty acids (SCFA), such as lactic acid, acetic acid, propionic acid, and butyric acid [22].

Our previous study demonstrated that CFS of probiotics containing SCFA has a skin barrier function in keratinocytes [23]. In this study, we investigated the skin protective effect of *Latilactobacillus sakei* wikim0066 (wikim0066) isolated from kimchi against UVB-irradiated skin fibroblast and animal models.

## 2. Materials and Methods

### 2.1. Preparation of Wikim0066 Samples

The wikim0066 was supplied by World Institute of Kimchi (Gwangju, Republic of Korea). The wikim0066 was identified by 16S rRNA gene sequencing and registered at the Korean Agricultural Culture Collection (KACC, Suwon, Republic of Korea) as *Latilactobacillus sakei* KACC 92194P. The strain was cultured in MRS broth (Difco, Sparks, MD, USA) at 37 °C for 24 h. Cultures were adjusted to an OD_600_ of 1.0 (10^8^–10^9^ CFU/mL) and subcultured for 18 h. To prepare CFS, the strain was centrifuged at 4000× *g* for 10 min at 4 °C, and the supernatant was collected. The supernatant was filtered and sterilized using a 0.22 μm polytetrafluoroethylene membrane filter (ADVANTEC, Tokyo, Japan) and used as a sample in an in vitro test.

For in vivo assays, wikim0066 was prepared as previously described [3]. The grown wikim0066 pellets were mixed well with a cryoprotectant mixture and freeze-dried (LP20-XXX, ilShin Biobase Co., Ltd., Gyeonggi-do, Republic of Korea). The powdered cells were harvested and mixed with maltodextrin to obtain a dilution to a cell density of 1 × 10^11^ CFU/g.

### 2.2. Cell Culture and UVB Treatment

Human foreskin fibroblasts (Hs68, ATCC, Manassas, VA, USA) were grown in Dulbecco’s modified eagle’s medium (DMEM, Gibco, Grand Island, NY, USA) supplemented with 10% fetal bovine serum (FBS, Gibco) and 1% penicillin-streptomycin (P/S, Gibco) at 37 °C in 5% CO_2_ atmosphere. UVB treatment was performed according to a previous report [24]. Hs68 cells were plated at 1.0 × 10^5^ cells/mL in the culture area, overnight. The cells were pretreated with 10% CFS of wikim0066 in the culture media for 24 h. Then, cells were washed once with Dulbecco’s phosphate-buffered saline (DPBS, Welgene, Daegu, Republic of Korea) and exposed to 30 mJ/cm^2^ of UVB using a UV crosslinker (PCL-1000, BoTeck, Gunpo, Republic of Korea) without the culture plate cover.

### 2.3. Cytotoxicity

Cytotoxicity was investigated using the MTT assay. Followed by UVB treatment as indicated in Section 2.2, cells were treated with 10% CFS of wikim0066 for 24 h. An MTT solution (0.1 mg/mL, Sigma-Aldrich, St. Louis, MO, USA) was added for 2 h. The formazan product was dissolved in dimethyl sulfoxide, and the absorbance was measured using a microplate reader (EPOCH2, BioTek, Winooski, VT, USA) at 550 nm.

### 2.4. Measurement of Type I Procollagen Production

Type I procollagen was measured by the type I procollagen kit (MK101, Takara, Shiga, Japan). Briefly, Hs68 cells were pretreated with 10% CFS of wikim0066 for 24 h followed by UVB treatment as indicated in Section 2.2, and Hs68 cells were treated with 10% CFS of wikim0066 for an additional 24 h. For the measurement of type I procollagen, the cell culture medium was collected and evaluated at 450 nm using a microplate reader (BioTek). 

### 2.5. Analysis of Short-Chain Fatty Acids (SCFAs) in CFS

SCFAs in CFS were analyzed by gas chromatography–mass spectrometry (GC–MS, QP2020 NXW/ORP230; Shimadzu, Kyoto, Japan) using a headspace solid-phase microextraction (SPME) method. Separation was carried out using a Stabilwax-DA column (60 m × 0.32 mm × 0.25 μm, Shimadzu), with high-purity helium as the carrier gas at a constant flow rate of 2 mL/min. GC analysis used splitless mode, with an oven temperature maintained at 50 °C for 2 min, increased to 200 °C at 10 °C per minute, and maintained for 5 min. The ionization source and quadrupole temperatures were 200 °C and 150 °C each. The electron energy was 70 eV. The MS analysis was confirmed in the scan range from 40 to 200 *m*/*z*, with a scan speed of 0.2 s per scan. The pure compounds were injected for qualitative analysis to compare retention time and MS data. Quantitative analysis was performed using the calibration curve of target compounds. The selected ion monitor (SIM) mode was applied in the electron impact mode at 65 eV.

### 2.6. Animals and Experimental Design

Female, HR-1 hairless, five-week-old mice were purchased from Central Lab Animal, Inc. (Seoul, Republic of Korea). The mice were maintained in climate-controlled quarters (22 ± 3 °C, 55 ± 15% humidity) with a light (12/12 h, light/dark) cycle. All experimental procedures (registration number: 2022-0017) were approved by the Committee on the Care of Laboratory Animal Resources at Chemon Co., Ltd. Mice were acclimatized to house conditions for 8 days and divided into three groups (*n* = 6/group): A normal group, a UVB-only treatment group, and a UVB with wikim0066 treatment group. The control and UVB-only treatment groups were orally administered 100 µL PBS without strain. The wikim0066 treatment group was orally administered 100 µL of wikim0066 (1 × 10^9^ CFU/head in PBS solution) for 12 weeks.

### 2.7. Mouse UVB Irradiation

UVB radiation was applied to the backs of the mice three times per week for 12 weeks using a UVB lamp (TL20W; Philips, Amsterdam, Netherlands). The starting dose of UVB radiation was 60 mJ/cm^2^ during the first 2 weeks, and the dose was increased to 120 mJ/cm^2^ for 3 weeks, 180 mJ/cm^2^ for 4 weeks, and finally, 240 mJ/cm^2^, which was maintained for 12 weeks. Skin replica preparation was performed during the 12th week of radiation exposure.

### 2.8. Evaluation of Skin Wrinkle Formation

After the mice were sacrificed at 12 weeks of the experiment, skin replicas were cast on the dorsal skin surface using Visioline VL-650 (Courage & Khazaka electronic GmbH, Cologne, Germany). The parameters for assessing skin wrinkles were the total wrinkle area, mean length, mean depth, and maximum wrinkle depth.

### 2.9. Histological Analysis

Dorsal skin specimens were obtained and fixed in 10% paraformaldehyde overnight, and fixed skin tissues were prepared as a paraffin-embedded block. The tissue slices were stained with hematoxylin and eosin (H&E) for the skin layers, examined using an optical microscope (BX61, Olympus, Tokyo, Japan), and photographed (DP80, Olympus).

### 2.10. Measurement of Transepidermal Water Loss (TEWL)

The TEWL of the dorsal skin was assessed using a GPSKIN barrier light (GPskin, Seoul, Republic of Korea) prior to and then at 0, 6, and 12 weeks after UVB exposure to determine epidermal water content. A GPSKIN barrier light (GPskin) was used for analysis to measure TEWL at the same time points before and after UVB irradiation.

### 2.11. Measurement of Skin Elasticity

Skin elasticity was measured using a Cutometer MPA580 (Courage and Khazaka Electronic GmbH). The Cutometer MPA580 was pressed onto the skin surface, and the skin deformation curves obtained were analyzed with the Win Cutometer MPA software to obtain the values of skin-elasticity parameters: R7 (the ratio of elastic recovery to all deformations).

### 2.12. Quantitative Real-Time Polymerase Chain Reaction (qRT-PCR) Analysis

Total RNA from Hs68 cells and dorsal skin tissue were isolated using the NucleoZOL reagent (MACHEREY-NAGEL, Dueren, Germany) according to the manufacturer’s instructions. RNA was synthesized using a cDNA reverse transcriptase premix (iNtron Biotechnology, Gyeonggi-do, Republic of Korea). qRT-PCR (Bio-Rad, Hercules, CA, USA) was performed using the amfiSure qGreen qPCR Master Mix (GenDEPOT, Katy, TX, USA). The PCR primers used for this analysis are listed in Table 1.

### 2.13. Protein Extraction and Western Blot

Whole fibroblast lysates and dorsal skin tissue were prepared in a radioimmunopre-cipitation assay (RIPA) buffer containing protease and phosphatase inhibitor cocktail (GenDEPOT). Cell lysates containing equal amounts of total protein, as determined using the Bradford assay (GenDEPOT), were prepared following the manufacturer’s instructions. The proteins were mixed with a sample buffer (iNtron). Western blotting was performed using 10% SDS-PAGE followed by protein transfer to polyvinylidene fluoride membranes (0.45 μm, Millipore, Darmstadt, Germany). The membrane was blocked with fast blocking buffer (Biomax, Seoul, Republic of Korea) for 5 min and washed three times with the TBST buffer. The membrane was incubated with the primary antibody (1:1000 dilution) containing 5% bovine serum albumin overnight at 4 °C, washed three times, and treated with the secondary antibody (1:5000 dilution) at room temperature for 1 h. All primary (β-actin, MMP-1, -3, protein cFos (cFos), transcription factor Jun (cJun), p-cFos, and p-cJun mouse monoclonal or extracellular signal-regulated kinase (ERK), cJun *N*-terminal kinase (JNK), p38 mitogen-activated protein kinases (p38), p-ERK, p-JNK, p-p38, p-NF-κB p65, and NF-κB p65 rabbit monoclonal) and secondary (anti-rabbit and anti-mouse IgG, horseradish peroxidase (HRP)-linked) antibodies were purchased from GenDEPOT and Cell Signaling Technology (Beverly, MA, USA). Protein bands were visualized using a LuminoGraph III Lite (ATTO, Tokyo, Japan) with the West-Q Femto Clean ECL solution (GenDEPOT). Quantitative analysis was conducted using ImageJ software (version 1.52a for Windows; NIH, Rockville, MD, USA).

### 2.14. Statistical Analysis

All experimental results are expressed as mean ± standard error of the mean (SEM) of three independent measurements. Statistical analysis was performed using Student’s *t*-test using Prism 5.02 (GraphPad Software, San Diego, CA, USA). Statistical significance was set at *p* < 0.05.

## 3. Results

### 3.1. Effect of CFS from Wikim0066 on Type I Procollagen Levels in UVB-Irradiated Hs68 Cells

**Figure 1:**
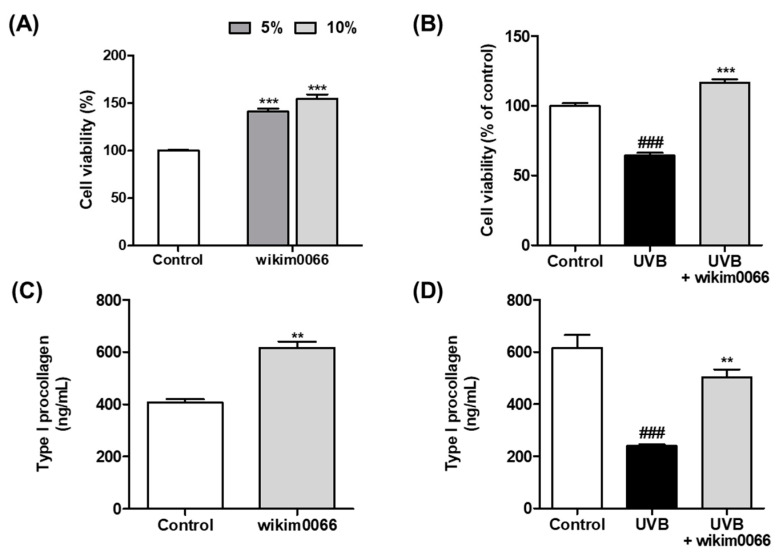
Effects of CFS from wikim0066 on cell viability and type I procollagen in UVB-irradiated Hs68 cells. Cell viability of only CFS treatment (**A**), CFS treatment with UVB irradiation (**B**), production of type I procollagen of only CFS treatment (**C**), and CFS treatment with UVB irradiation (**D**). Cultured Hs68 cells were pre-treated with 10% CFS for 24 h. After UVB (30 mJ/cm^2^) irradiation, cells were immediately treated with fresh medium containing 10% CFS for 24 h. Cell viability was confirmed by MTT assay. The results are expressed as mean ± SEM (*n* = 3). ^###^ *p* < 0.001 vs. control and ** *p* < 0.01 and *** *p* < 0.001 vs. UVB-control.

**Figure 2:**
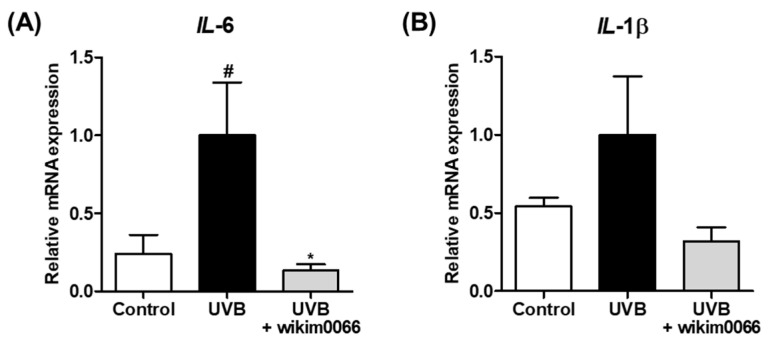
Effect of CFS from wikim0066 of *IL-6* (**A**) and *IL-1β* (**B**) mRNA expression on UVB-irradiated Hs68 cells. Cells were pretreated with 10% CFS of wikim0066 and irradiated UVB (30 mJ/cm^2^), then treated with 10% CFS of wikim0066 for 3 h. The mRNA expression was normalized to that of GAPDH and presented as the mean ± SEM (*n* = 3). ^#^ *p* < 0.05 vs. control and * *p* < 0.05 vs. UVB-control.

We confirmed the photoprotective effect of wikim0066 on UVB-irradiated Hs68 cells using the MTT assay. When treated with 5 and 10% CFS in Hs68 cells without UVB, the cell viability increased by more than 50% compared to the control group (*p* < 0.001, Figure 1A). In Figure 2B, UVB irradiation reduced cell viability by 64.3 ± 3.2% compared to the control (*p* < 0.001). However, the viability of CFS-treated cells was restored to control levels (>100%) compared to the UVB control.

We also evaluated the effects of CFS on type I procollagen production. The production of type I procollagen in cells treated with CFS without UVB was significantly increased compared to that in the control (*p* < 0.01, Figure 1C). In addition, the production of type I procollagen, which was significantly reduced by UVB exposure, was significantly increased by CFS treatment (*p* < 0.01, Figure 1D).

### 3.2. Effect of CFS from Wikim0066 on Inflammatory Cytokines mRNA Expressions on UVB-Irradiated Hs68 Cells

The effect of CFS of wikim0066 on mRNA expression of inflammatory cytokines in UVB-irradiated Hs68 cells was evaluated. The mRNA expression of *IL-6* and *IL-1β* was increased under UVB exposure compared to that in the control, whereas wikim0066 treatment significantly reduced the mRNA expression of *IL*-*6* (inhibition rate, 86.6 ± 4.1%, *p* < 0.05) and *IL-1β* (inhibition rate, 68.2 ± 9.2%) (Figure 2A,B).

### 3.3. Effect of CFS from Wikim0066 on mRNA and Protein Expressions of MMP-1 and MMP-3 on UVB-Irradiated Hs68 Cells

**Figure 3:**
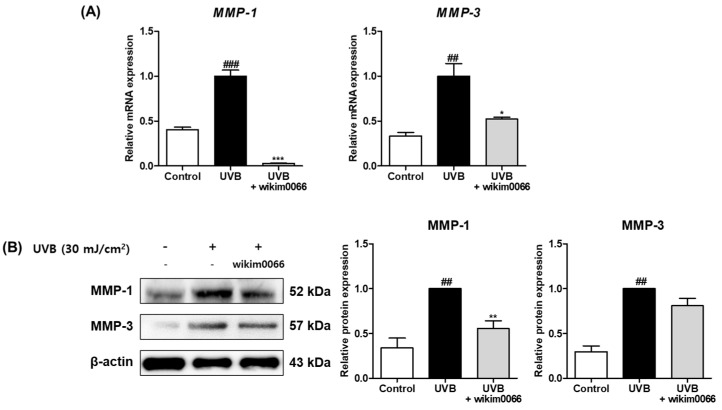
Effect of CFS from wikim0066 on mRNA expressions (**A**) and protein expressions (**B**) of *MMP-1* and *MMP-3* in UVB-irradiated Hs68 cells. Cells were pretreated with 10% CFS and irradiated with UVB (30 mJ/cm^2^), then treated with fresh culture medium containing 10% CFS for 48 h. The mRNA intensities were normalized to that of GAPDH, and proteins were normalized to that of β-actin. Results are presented as the mean ± SEM (*n* = 3). ^##^ *p* < 0.01, and ^###^ *p* < 0.001, vs. control and * *p* < 0.05, ** *p* < 0.01, and *** *p* < 0.01 vs. UVB-control.

To assess the effects of modulating MMP expression by CFS from wikim0066, mRNA expression of *MMP-1* and *-3* in UVB-irradiated Hs68 cells was measured by qRT-PCR. The mRNA expression of *MMP-1* (*p* < 0.001) and *MMP-3* (*p* < 0.01) was significantly increased in the UVB irradiation group compared to t in those in the control group (Figure 3A). In contrast, the mRNA expression of *MMP-1* (inhibition rate, 97.1 ± 0.4%, *p* < 0.001) and *MMP-3* (inhibition rate, 47.8 ± 2.5%, *p* < 0.05) was significantly decreased by wikim0066 treatment compared to UVB-control.

In addition, we investigated the effects of wikim0066 on protein expressions of MMP-1 and -3 in UVB-irradiated Hs68 cells using Western blotting. The protein expression of MMP-1 (*p* < 0.01) and MMP-3 (*p* < 0.01) was significantly increased in the UVB irradiation group compared to those in the control group (Figure 3B). In contrast, MMP-1 (inhibition rate, 44.6 ± 7.2%, *p* < 0.01) and MMP-3 (inhibition rate, 58.1 ± 13.6%) protein expression was decreased in wikim0066-treated cells compared to the UVB-control.

### 3.4. Effect of CFS from Wikim0066 on MAPK and AP-1 Signaling Pathway on UVB-Irradiated Hs68 Cells

**Figure 4:**
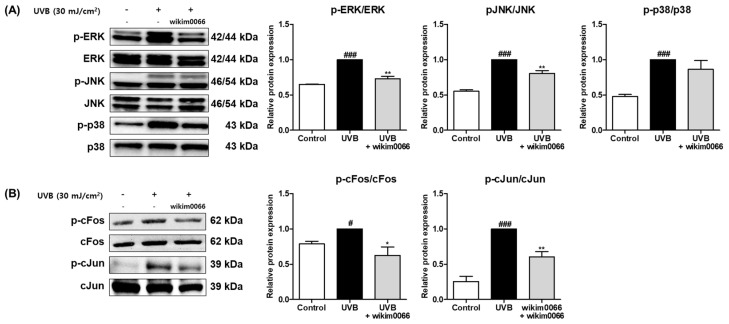
Effect of CFS from Wikim0066 on MAPK (**A**), and AP-1 (**B**) signaling pathway in UVB-irradiated Hs68 cells. Cells were pretreated with 10% CFS of wikim0066 and irradiated with UVB (30 mJ/cm^2^), then treated with 10% CFS of wikim0066 for 1 h. The protein level was normalized to that of β-actin. Results are presented as the mean ± SEM (*n* = 3). ^#^ *p* < 0.05, and ^###^ *p* < 0.001 vs. control, and * *p* < 0.05, and ** *p* < 0.01 vs. UVB-control.

We investigated whether CFS from wikim0066 is involved in the MAPK and AP-1 signal pathways. In Figure 4A, the protein expression of p-ERK, p-JNK, and p-p38 was significantly increased by UVB treatment (Figure 4A, *p* < 0.001). However, CFS treatment significantly reduced the phosphorylation of ERK (inhibition rate, 27.0 ± 3.3%, *p* < 0.01) and JNK (inhibition rate, 19.6 ± 4.1%; *p* < 0.01). Phosphorylation of p38 did not show a significant decrease.

In addition, UVB irradiation induced the protein expression of cFos (*p* < 0.05) and cJun (*p* < 0.001), AP-1 subunits (Figure 4B). However, CFS treatment significantly reduced the phosphorylation of cFos (inhibition rate, 38.4 ± 12.1%, *p* < 0.05) and cJun (inhibition rate, 39.7 ± 7.5%, *p* < 0.01) in UVB-irradiated cells.

### 3.5. Production of SCFAs in CFS from Wikim0066

To confirm the production of SCFAs in CFS of wikim0066, GC/MS analysis was performed (Table 2). The ion (*m*/*z*) of acetic, propionic, and butyric acid was confirmed at 60, 45, 43 *m*/*z*, 74, 73 *m*/*z*, 60, 73, and 42 *m*/*z*, and the retention time was 12.42, 13.51, and 14.70 min, respectively. In the CFS of wikim0066, acetic acid was the highest content (3.20 ± 0.15 mg/g) among SCFA. Next, butyric acid (4.04 ± 0.67 μg/g) and propionic acid (1.29 ± 0.15 μg/g) were produced in order, respectively.

### 3.6. Effect of Wikim0066 Administration on Wrinkle-Related Parameters on UVB-Irradiated HR-1 Hairless Mice Model

**Figure 5:**
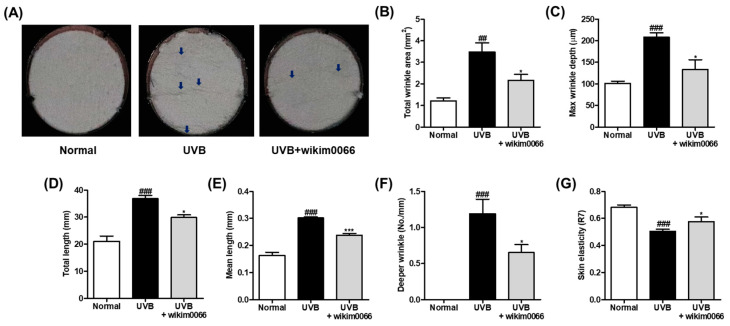
Effect of wikim0066 administration on wrinkle-related parameters in UVB-irradiated hairless mice. Skin replica (**A**), total wrinkle area (**B**), max wrinkle depth (**C**), total length (**D**), mean length (**E**), and deeper wrinkle (**F**) were measured on dorsal skin replica. The blue arrows indicate the wrinkled area. Skin elasticity (**G**) was measured using Cutometer. HR-1 hairless mice were orally administrated wikim0066 (1 × 10^9^ CFU/head) three times a week for 12 weeks with UVB irradiation. Results are expressed as mean ± SEM (*n* = 6). ^##^ *p* < 0.01, ^###^ *p* < 0.001 vs. normal, and * *p* < 0.05, *** *p* < 0.001 vs. UVB-control.

The degree of wrinkle formation was confirmed by observing the photographs of the skin replica. UVB irradiation promotes wrinkle formation on the dorsal skin (Figure 5A). We examined wrinkle-related parameters to verify the photoprotective effect of wikim0066 on UVB-irradiated HR-1 hairless mice. As a result, the wrinkle-related parameters, including total wrinkle area (*p* < 0.01), mean length, maximum depth, total length, mean length, deeper wrinkles, and skin elasticity (*p* < 0.001) were significantly increased by UVB irradiation (Figure 5B–G). In contrast, wikim0066 administration significantly reduced all wrinkle-related parameters tested (*p* < 0.05), including mean length (*p* < 0.001).

### 3.7. Effect of Wikim0066 Administration on Epidermal Thickness and TEWL on UVB-Irradiated HR-1 Hairless Mice Model

**Figure 6:**
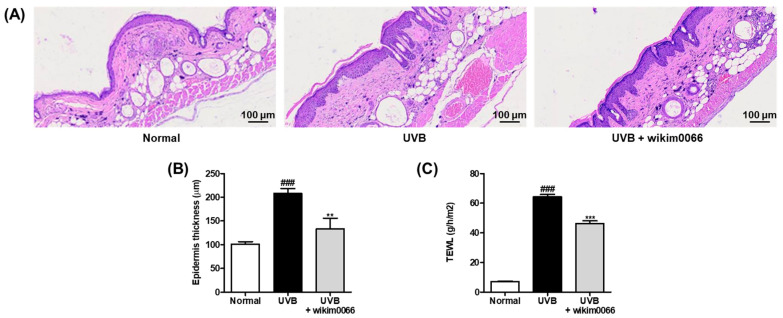
Effect of wikim0066 administration on histological representative image of dorsal skin section (**A**), epidermis thickness (**B**), and TEWL (**C**) in UVB-irradiated hairless mice. Epidermal thickness was measured by H&E staining using a microscope at 100 × magnification. HR-1 hairless mice were orally administrated wikim0066 (1 × 10^9^ CFU/head) three times a week for 12 weeks with UVB irradiation. Results are expressed as mean ± SEM (*n* = 6). ^###^ *p* < 0.001 vs. normal, and ** *p* < 0.01, *** *p* < 0.001 vs. UVB-control.

To evaluate the changes in the epidermal thickness of dorsal skin after UVB irradiation in mice, H&E staining was performed (Figure 6A). UVB irradiation significantly increased the thickening of the dorsal skin of the UVB group compared to that of the normal group (*p* < 0.001). In contrast, wikim0066 administration diminished the epidermis thickness compared to that in the UVB group (*p* < 0.01) (Figure 6B).

We also evaluated TEWL as a skin moisture-related factor in the UVB-irradiated HR-1 mice. UVB irradiation significantly increased the TEWL in the UVB group compared to that in the normal group (*p* < 0.001). However, wikim0066 administration reduced TEWL compared with that in the UVB group (*p* < 0.001) (Figure 6C).

### 3.8. Effect of Wikim0066 Administration on Inflammatory Cytokines mRNA and NF-κB Expressions on UVB-Irradiated HR-1 Mice Model

**Figure 7:**
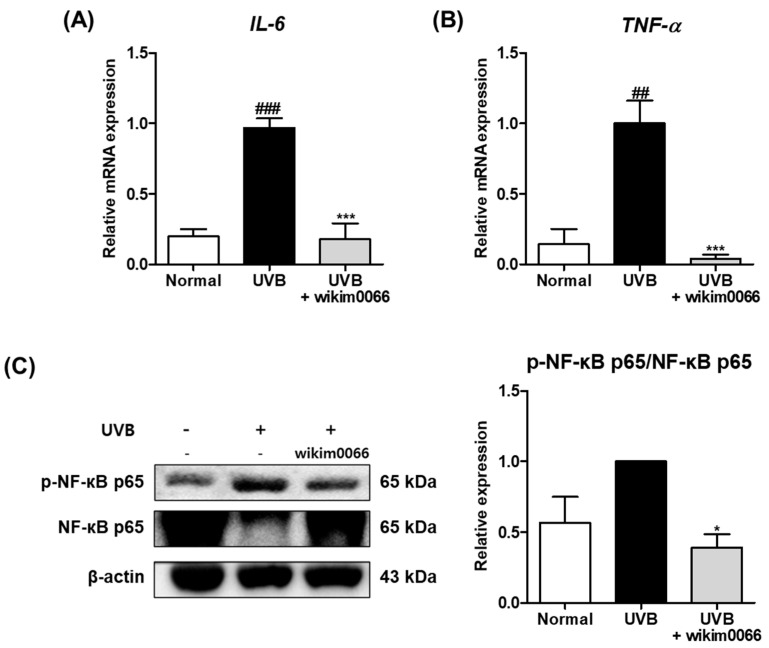
Effect of wikim0066 administration on mRNA expressions of *IL-6* (**A**), *TNF-α* (**B**), and protein expression of NF-κB p65 (**C**) in UVB-irradiated hairless mice. HR-1 hairless mice were orally administrated wikim0066 (1 × 10^9^ CFU/head) three times a week for 12 weeks with UVB irradiation. The mRNA level was normalized to that of GAPDH, and protein was normalized to that of β-actin. Results are presented as the mean ± SEM (*n* = 6). ^##^ *p* < 0.01, ^###^ *p* < 0.001 vs. normal and * *p* < 0.05, *** *p* < 0.001 vs. UVB-control.

The effect of wikim0066 administration on mRNA expression of inflammatory cytokines was evaluated in UVB-irradiated HR-1 mice. The *IL-6* (*p* < 0.001) and *TNF-α* (*p* < 0.01) mRNA expression levels were significantly increased by UVB irradiation compared to the normal group (Figure 7A,B), while wikim0066 administration significantly reduced *IL-6* (inhibition rate, 96.0 ± 2.9%, *p* < 0.001) and *TNF-α* (inhibition rate, 82.1 ± 11.4%, *p* < 0.001) mRNA expression.

We also evaluated the protein expression of NF-κB p65 in the UVB-irradiated HR-1 mice. The protein expression of p-NF-κB p65 was increased in the UVB group (Figure 4C). However, wikim0066 administration significantly reduced the phosphorylation of NF-κB p65 (inhibition rate, 61.8 ± 9.3%, *p* < 0.05).

### 3.9. Effect of Wikim0066 Administration on MMP-1, -3, MAPK and AP-1 Signaling Pathway on UVB-Irradiated HR-1 Mice Model

**Figure 8:**
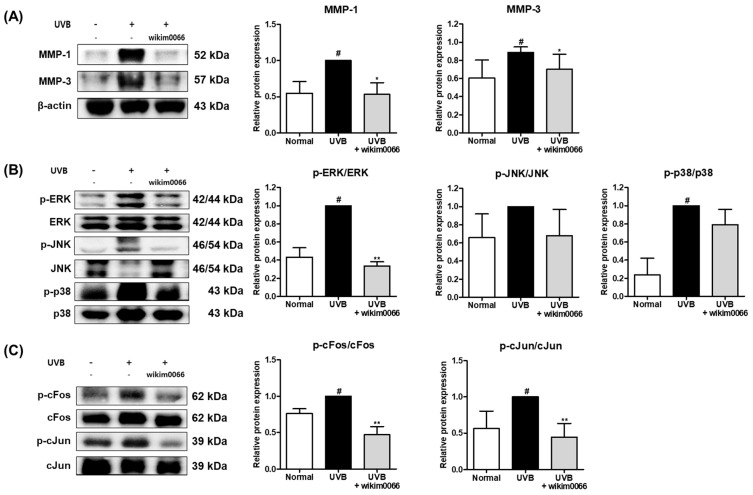
Effect of wikim0066 administration on MMP-1 and -3 (**A**), MAPK (**B**), and (**C**) AP-1 protein expression in UVB-irradiated HR-1 hairless mice. HR-1 hairless mice were orally administrated wikim0066 (1 × 10^9^ CFU/head) three times a week for 12 weeks with UVB irradiation. The protein level was normalized to that of β-actin. Results are presented as the mean ± SEM (*n* = 6). ^#^ *p* < 0.05 vs. normal and * *p* < 0.05, ** *p* < 0.01 vs. UVB-control.

The protein expression of MMP-1 and MMP-3 was significantly increased in UVB-irradiated HR-1 mice (*p* < 0.05). However, wikim0066 administration reduced the expression of MMP-1 (46.9 ± 16.1%) and MMP-3 (42.5 ± 14.3%) in UVB-irradiated HR-1 mice (*p* < 0.05) (Figure 8A).

UVB irradiation also significantly induced the expression levels of p-ERK and p-p38 (*p* < 0.05). However, wikim0066 administration significantly reduced the phosphorylation of ERK (66.5 ± 3.7%) in UVB-irradiated HR-1 mice (*p* < 0.05) (Figure 8B).

In addition, UVB irradiation significantly induced the protein expression levels of cFos and cJun (*p* < 0.05). However, wikim0066 administration significantly reduced the phosphorylation of cFos (38.4 ± 12.1%) and cJun (39.7 ± 7.5%) in UVB-irradiated HR-1 mice (*p* < 0.01) (Figure 8C).

## 4. Discussion

Although many studies have reported the interaction of probiotics with the skin, the mechanism remains unclear. Previous studies have reported that oral administration of probiotics, especially *Lactobacillus*, can improve wrinkles, including skin barrier function [25]. *Lactobacillus acidophilus* KCCM12625P regulates wrinkle-related genes in human skin cells [26]. *Limosilactobacillus reuteri* DSM 17938 regulates UVB-induced inflammation and skin barrier function in a human ex vivo model [27]. *Lactiplantibacillus plantarum* HY7714 inhibits UVB-induced wrinkle-related factors in human skin cells and animal models [28]. *Lacticaseibacillus rhamnosus* KCTC5033 improves hydration in human facial skin [29]. Therefore, in this study, we confirmed the effects of wikim0066 on skin aging and related pathways in UVB-irradiated Hs68 cells and an animal model.

Probiotics indirectly protect the skin by regulating tight junctions in the intestinal epithelium, thereby reducing the penetration of inflammatory cytokines into blood vessels [24]. Recent studies have reported that gut flora affects the skin [22]. The gut microbiome affects the skin by regulating the function and composition of the innate or adaptive immune system [30]. In addition, metabolites such as SCFAs including acetic, butyric, and propionic acid produced by gut microbes can be released into the bloodstream and act on distant organs and systems such as the skin [16]. Butyric acid, the major SCFA in the human intestine, has been reported to mediate FFAR2 to regulate the production of UVB-induced pro-inflammatory cytokines on the dorsal skin of mice [31]. Moreover, propionic acid has been reported to inhibit the pigmentation induced by UVB [32]. Compared with the SCFA content of other Lactobacillus genera suggested in our previous study, wikim0066 showed a high level of butyric acid content (Table 2) [33]. Therefore, these results suggest that wikim0066 may have inhibited the expression of UVB-induced pro-inflammatory cytokines.

Inflammatory cytokines can also affect skin aging [26]. UVB has been reported to stimulate inflammatory cytokines, such as IL-6, IL-1β, and TNF-α in fibroblasts [34]. TNF-a and IL-1β share several properties. TNF-a and IL-1β can stimulate IL-6 synthesis in several cell types, further causing secondary inflammatory effects [35]. IL-6 then mediates the effects of TNF-α and IL-1β to amplify the inflammatory response through a cytokine cascade with overlapping properties, leading to an acute-phase response [36]. Our results showed that wikim0066 reduced the mRNA expression of pro-inflammatory cytokines in UVB irradiation in vitro and in vivo. In the inflammatory response, TNF-α stimulates the activation of NF-κB and AP-1 and the secretion of IL-1β. Stimulated IL-1β, similar to TNF-α, activates NF-κB and AP-1 [37]. NF-κB is a transcription factor that plays a role in MMP expression in UVB-irradiate skin [38]. AP-1 is composed of the subunits cFos and cJun and acts as an important transcription factor in UVB-irradiated skin [10]. In our study, NF-κB p65 was activated by UVB irradiation, whereas wikim0066 inhibited the phosphorylation of NF-κB p65 in UVB-irradiated in vitro and in vivo. In addition, wikim0066 prevented the phosphorylation of cFos and cJun upon UVB irradiation in vitro and in vivo.

UVB irradiation accelerates aging of the skin epidermis and decreases collagen levels, resulting in the appearance of wrinkles and a loss of elasticity through a decrease in ECM components [39]. Our study confirmed the wrinkle area, depth, length, and number of wrinkles through dorsal skin replicas, which were reduced by wikim0066. In addition, skin elasticity was restored by wikim0066. Epidermal thickness was reduced by wikim0066. Skin is a barrier that protects the body and prevents water loss [40]. UVB exposure has been reported to increase TEWL by damaging the dermis [41]. In the present study, wikim0066 markedly prevented TEWL in UVB-irradiated HR-1 hairless mice.

Collagen, a major component of the ECM, is composed of 28 types of collagens and has been identified in various tissues such as tendons, ligaments, skin, and teeth. Type I collagen, which is mostly distributed in the human body, is especially found in the skin [42]. In dermal fibroblasts, procollagen migrates to the extracellular space and is synthesized into type I collagen polypeptide chains [1]. In our study, wikim0066 markedly restored the production of type I procollagen on UVB-irradiated Hs68 cells.

MMP-1 and -3, the major contributors to type I collagen breakdown, are upregulated by NF-κB and AP-1 [10]. In the present study, wikim0066 inhibited the mRNA and protein expression of MMP-1 and MMP-3. MAPK, including ERK, JNK, and p38, not only leads to the activation of UVB-irradiated NF-κB but also leads to the activation of AP-1, which consequently increases the expression of MMP [38]. UVB stimulated the activation of MAPK in skin cells and animal models; however, wikim0066 markedly reduced the phosphorylation of MAPK. As mentioned above, the expression of NF-κB and AP-1 was decreased by wikim0066. In this study, wikim0066 improved collagen, moisture, skin barrier, and wrinkle formation by regulating MMP-1 and -3 expressions via the MAPK/AP-1/NF-κB signaling pathway in vitro and in vivo. Although the effects of wikim0066 have been confirmed in vitro and in vivo, clinical studies on its skin protection effect against UVB irradiation are required.

## 5. Conclusions

**Figure 9:**
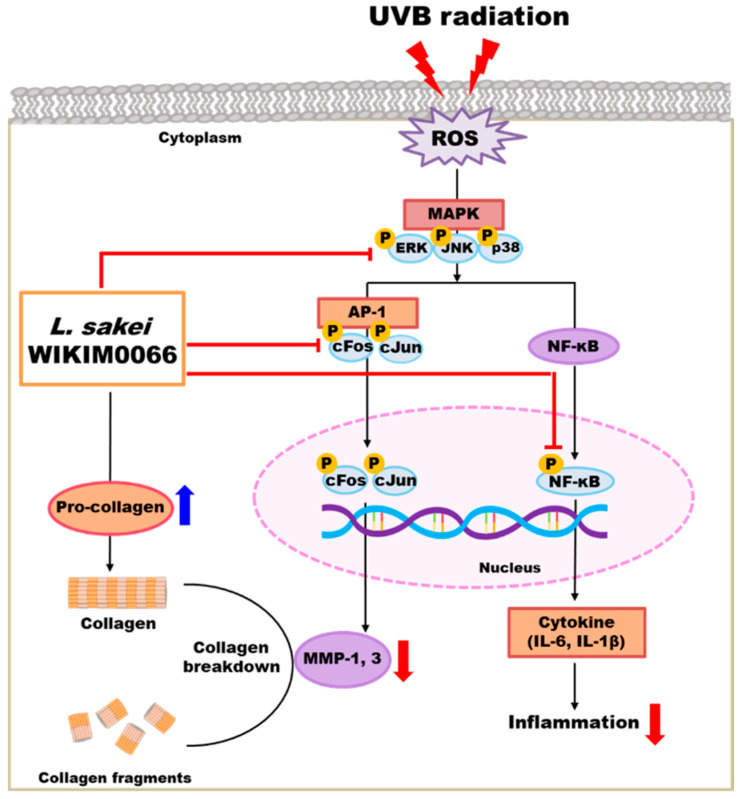
Schematic diagram of the effects of wikim0066 on UVB-irradiated skin cells and animal model.

We confirmed that wikim0066 restores the production of type I procollagen in UVB-irradiated Hs68 cells. Moreover, wikim0066 reduced the mRNA and protein expression of wrinkle-related factors in UVB-irradiated Hs68 cells and HR-1 hairless mice. In conclusion, wikim0066 improved collagen, moisture, skin barrier, and wrinkle reduction by regulating the expression of MMP-1 and MMP-3 via the MAPK/AP-1/NF-κB signaling pathway in vitro and in vivo (Figure 9). Therefore, wikim0066 is a potential probiotic candidate for skin aging and should be verified in future clinical studies.

## Figures and Tables

**Table 1:** Primer sequences for qRT-PCR analysis.

	Gene	Primer Sequence	
In vitro	*MMP-1*	F ^1^	GCATATCGATGCTGCTCTTTC
		R ^2^	GATAACCTGGATCCATAGATCGTT
	*MMP-3*	F	CAAAACATATTTCTTTGTAGAGGACAA
		R	TTCAGCTATTTGCTTGGGAAA
	*IL-6*	F	AAGCCAGAGCTGTGCAGATGAGTA
		R	TGTCCTGCAGCCACTGGTTC
	*IL-1β*	F	AGAAGTACCTGAGCTCGCCA
		R	CCTGAAGCCCTTGCTGTAGT
	*GAPDH*	F	ACCCACTCCTCCACCTTTG
		R	ACCCACTCCTCCACCTTTG
In vivo	*IL-6*	F	GAGACTTCCATCCAGTTGCCT
		R	TGGGAGTGGTATCCTCTGTGA
	*TNF-* *α*	F	TGTCTACTCCTCAGAGCCCC
		R	TGAGTCCTTGATGGTGGTGC
	*GAPDH*	F	TCTCCCTCACAATTTCCATCC
		R	GGGTGCAGCGAACTTTATTG

^1^ F, forward; ^2^ R, reverse; *MMP*, matrix metalloproteinase; *IL*, interleukin; *GAPDH,* glyceraldehyde 3-phosphate dehydrogenase; *TNF*, tumor necrosis factor.

**Table 2:** Quantitative analysis data of SCFAs in wikim0066.

Sample	Acetic Acid (mg/g)	Propionic Acid (μg/g)	Butyric Acid (μg/g)
wikim0066	3.20 ± 0.58	1.29 ± 0.15	4.04 ± 0.67

All data are presented as the mean ± SEM (*n* = 3).

## Data Availability

Not applicable.
